# Heat-associated hyponatremia and its outcomes in very old inpatients in northern Germany

**DOI:** 10.1007/s00391-026-02635-5

**Published:** 2026-09-02

**Authors:** Naomi Mader, Stefan Muthers, Klaus Albrecht, Martin Scharpenberg, Andreas Kribben, Stefan Herget-Rosenthal

**Affiliations:** 1 Department of Nephrology, University Duisburg-Essen, Duisburg-Essen, Germany; 2 Department of Medicine, Rotes Kreuz Krankenhaus, Bremen, Germany; 3 Research Centre for Human Biometeorology, Deutscher Wetterdienst, Freiburg im Breisgau, Germany; 4 Competence Center for Clinical Trials—Biometry, University of Bremen, Bremen, Germany; 5 Universitätsmedizin Essen, University Duisburg-Essen, Essen, Germany

**Keywords:** Climate, Heat, Electrolyte disorder, Outcome, Temperature, Klima, Hitze, Hyponatriämie, Outcome, Temperatur

## Abstract

**Background:**

Hyponatremia is frequent in very old patients ≥ 80 years (VOP). Data for hyponatremia associated with moderate heat in VOP are scarce. We aimed to provide epidemiological data, to identify risk factors of hyponatremia and to present outcomes in VOP after hot and cold days in northern Germany.

**Methods:**

Of 22,085 VOP admitted unplanned to the departments of internal medicine and trauma surgery in 2011–2023, we studied those admitted 1–2 days after hot (*N* = 308) and cold days (*N* = 253) in June-August of these years (57.9% female, median age 86 years). Heat was defined as daily mean temperatures ≥ 95th (24.4 °C), cold as ≤ 5th percentile (12.3 °C) in June-August from 2011–2023. Primary outcome was hyponatremia on admission. Secondary outcomes were risk factors of hyponatremia and the frequency of major adverse hyponatremia events until day 30 (MAHE30, intensive care unit and/or long hospital stay, mortality and/or discharge to a care facility).

**Results:**

In 561 patients, hyponatremia occurred more often after hot than cold days (36.7% vs. 15.0%; *P* < 0.001). Hyponatremia was predominately mild, acute or acute on chronic and isotonic in patients admitted after both hot and cold days. Hyponatremia rates increased by 50% after 3 consecutive hot days. Heat was a strong risk factor of hyponatremia. In 151 hyponatremic patients, MAHE30 occurred more often after hot compared to cold days (55.6% vs. 31.6%; *P* = 0.008). Heat was identified as a strong risk factor also of MAHE30 in hyponatremic patients.

**Conclusion:**

Moderate heat in northern Germany is associated with an increased rate of hyponatremia and is a strong risk factor of hyponatremia in VOP. After hot days, hyponatremic VOP are at an increased risk for severe associated morbidity and mortality.

**Supplementary Information:**

The online version of this article (https://doi.org/10.1007/s00391-026-02635-5) contains supplementary material, which is available to authorized users.

## Abstract (de)

**Hintergrund:**

Hyponatriämie tritt häufig bei Hochbetagten (≥ 80 Jahre; „very old people“, VOP) auf. Daten zu Hyponatriämie bei moderater Hitze bei VOP sind selten. Wir erfassten die Rate von Hyponatriämie, deren Risikofaktoren und Auswirkungen auf VOP nach heißen und kalten Tagen in Norddeutschland.

**Methoden:**

Von 22.085 ungeplanten internistischen und unfallchirurgischen, stationären VOP schlossen wir diejenigen ein, die in den Monaten Juni bis August der Jahre 2011 bis 2023 jeweils 1–2 Tage nach heißen (*n* = 308) und kalten Tagen (*n* = 253) aufgenommen wurden (57,9 % Frauen, Median 86 Jahre). Hitze wurde definiert als Tagesmitteltemperaturen ≥ 95. Perzentile (24,4 °C), Kälte als ≤ 5. Perzentile (12,3 °C) zwischen Juni und August 2011 bis 2023. Primärer Endpunkt war die Hyponatriämie bei Aufnahme. Sekundäre Endpunkte waren Risikofaktoren der Hyponatriämie und die Häufigkeit von MAHE30 („major adverse hyponatraemia events“ bis Tag 30: Intensivstation, langer Krankenhausaufenthalt, Mortalität, und/oder poststationäre Verlegung in eine Pflegeeinrichtung).

**Ergebnisse:**

Bei 561 VOP trat Hyponatriämie häufiger nach heißen als nach kalten Tagen auf (36,7 vs. 15,0 %; *p* < 0,001). Die Hyponatriämie war hauptsächlich mild, akut oder akut-auf-chronisch und isotonisch sowohl nach Hitze als auch nach Kälte. Die Hyponatriämierate stieg um 50 %, wenn ≥ 3 heiße Tage aufeinander folgten. Hitze war ein starker Risikofaktor der Hyponatriämie. Bei 151 Patienten mit Hyponatriämie trat MAHE30 häufiger nach heißen als kalten Tagen auf (55,6 vs. 31,6 %; *p* = 0,008). Hitze wurde auch als starker Risikofaktor von MAHE30 bei Hyponatriämie identifiziert.

**Diskussion:**

Moderate Hitze in Norddeutschland ist bei VOP mit einer erhöhten Rate an Hyponatriämie assoziiert und stellt einen starken Risikofaktor hierfür dar. Nach heißen Tagen besteht für VOP mit Hyponatriämie ein erhöhtes Risiko für assoziierte Morbidität und Mortalität.

## Background

Hyponatremia is frequent and associated with substantially increased morbidity and mortality [1, 3, 9, 22]. Its incidence is especially high in old patients [3, 8, 15]. Physiological renal changes combined with a high prevalence of diabetes, hypertension and chronic kidney disease (CKD) lower the compensatory ability and cause excessive vulnerability for hyponatremia in the old [15]. In parallel, increased life expectancy has resulted in a rapid increase of Europeans aged 80 years and above, subsequently termed very old patients (VOP) [7]. Identification, modification or elimination of risk factors may prevent hyponatremia and reduce its severity or the severity and frequency of adverse outcomes [1, 3, 20]. Heat has been described as a risk factor of hyponatremia [10, 13, 16–18, 21]. Especially in Europe, heat and heat-related morbidity and mortality have substantially increased [5, 19]; however, there is no uniform definition of heat, previous studies predominately demonstrate the effect of substantial heat on hyponatremia and data on hyponatremia in VOP during moderate hot summers in Central Europe with a temperate oceanic climate and on hyponatremia-associated outcome after heat are scarce [10, 16–18, 21]. We thus aimed to (1) provide epidemiological data on hyponatremia, (2) identify risk factors and (3) present its outcomes, all in VOP, comparing the effect of moderately hot to cold temperatures during summers in northern Germany.

## Patients and methods

### Patients

This is a cohort study of all VOP admitted unplanned to the Departments of Medicine and Trauma Surgery, Red Cross Hospital (Rotes Kreuz Krankenhaus), Bremen, northern Germany, which is a secondary care hospital with 302 beds. Most patients with conditions potentially associated with hyponatremia, such as cognitive impairment, falls, gait disturbance, weakness and traumatic injuries were admitted unplanned to these departments. We included patients between 1 June and 31 August in each year from 2011–2023, if they were admitted after either hot or cold days. We grouped patients into the hot days group if they were admitted 1 or 2 full days after a hot day, e.g. 4 or 5 July when 3 July was a hot day. The same criteria also applied for cold days. Consistent with definitions of the German Weather Service (Deutsche Wetterdienst, DWD), we defined hot days as those days with daily mean temperatures (Tmean) exceeding the 95th percentile and cold days as those days with Tmean below the 5th percentile of all days during June–August from 2011–2023 and all measured in Bremen [19]. We chose June–August from 2011–2023 as this period included the highest number of days with maximum temperatures ≥ 30.0 °C. We also refer to temperatures in Bremen above the 95th percentile as moderate heat as high temperatures in northern Europe are more temperate than in tropical or subtropical areas. A lag of 1–2 days from the onset of pathophysiological effects triggered by heat up to admission had been found to be relevant [10]. The Tmeans were provided by the DWD Bremen weather station and 87.2% of our patients lived within a 6 km radius of this weather station.

Hot versus cold days were studied to permit comparison with previous studies which also used the ends of the temperature range and to highlight potential effects of temperature on hyponatremia [10, 13, 16, 21].

Patients were required to have blood glucose, serum creatinine and sodium (S-Na) measurements on admission. We excluded patients transferred from other hospitals, those with CKD stage G5 (*N* = 19), missing data on admission (*N* = 18), and decisions on admission to withhold life-sustaining treatment (*N* = 23) (flow diagram with details of study participants provided in Supplement Fig.). Most data were collected from electronic hospital records, some preadmission and follow-up data also from primary care physician’s records.

### Outcomes and variables

Primary outcome was hyponatremia on admission. Hyponatremia was defined as adjusted S‑Na < 135 mmol/L and subclassified (Suppl. Tab. 1) [1, 3, 9, 19, 20, 22]. The S‑Na was measured by direct potentiometry using blood gas analyzers in undiluted, whole blood and adjusted for blood glucose > 100 mg/dl [20]. Preadmission S‑Na and estimated glomerular filtration rate (eGFR) values were taken from the 3 months prior to admission and used to determine chronic hyponatremia and CKD (definitions presented in Suppl. Tab. 1). Baseline values were those recorded on admission. Besides heat, as the assumed principal cause of hyponatremia, hypovolemia and acute kidney injury, we considered ‘other causes of hyponatremia defined as diarrhea, excessive sweating, fever and vomiting in the 3 days prior to admission as potential further causes of hyponatremia. The following drugs were subsumed under “other drugs causing hyponatremia” when regularly administered prior to hospital admission, e.g., angiotensin-converting enzyme inhibitors, angiotensin-II receptor antagonists, anticonvulsants, antidepressants, neuroleptics and opioids [3, 12]. Correction of hyponatremia was subdivided into complete, partial, marginal and none (Suppl. Table 1). Further definitions are presented in Suppl. Table 1.

Secondary outcomes were risk factors of hyponatremia and the newly created variable major adverse hyponatremia events until day 30 after admission (MAHE30). Hyponatremia per se is of little relevance as an outcome unlike its adverse effects [1, 9, 20, 22]. The MAHE30 is a composite outcome of ≥ 1 clinically highly relevant adverse effect of hyponatremia: (1) admission to an intensive care unit (MAHE30^ICU^), (2) length of stay in hospital of all included VOPs admitted after hot or cold days > 90th percentile, (3) mortality and/or (4) discharge to a care facility in patients living at home prior to admission (MAHE30^Care facility^) [1, 9, 20, 22].

### Statistical analyses

Data were complete except for preadmission S‑Na and eGFR values which were available in 86.7% and 82.5%, respectively. In cases of multiple values available, we analyzed the value closest to that on hospital admission. In cases of missing values, these were not replaced with estimated values but we exclusively analyzed the existing data. Continuous variables were expressed as means ± SD or medians (25th; 75th percentile). Data from all patients and those exclusively with hyponatremia, both separated in the cohorts admitted after hot and cold days, were compared using unpaired Student’s t‑test or Wilcoxon-test. Categorical variables were expressed as total counts and percentages and compared with χ^2^, Fisher’s exact or Cochran-Armitage tests. We constructed multivariate, logistic regression models with hyponatremia as a dependent variable including all patients, MAHE30 and MAHE30^ICU^ as dependent variables, the latter two exclusively in patients with hyponatremia. We included all risk factors potentially associated with these variables with *P* < 0.10 in bivariate analysis (Suppl. Tables 2 and 3). These models were assessed using Hosmer-Lemeshow tests and C‑statistics. The two-sided *P* < 0.05 was considered statistically significant.

## Results

A total of 561 patients were studied, with the median age above 80 years, predominately female and approximately half received either home care or lived in care facilities (Table 1). The majority of patients were admitted after hot days (54.9%, *N* = 308) During the time period of 2011–2023, 60 days were defined as hot and 59 days as cold days. The number of hot and cold days per year varied from 0–12. On average each year had 4 cold and 5 hot days. In the summer of 2017 there were no hot days, as opposed to 2019 with the maximum number of 12 hot days. Median values of T_mean_ were 24.4 °C (interquartile ranges 23.9; 25.7 °C) on hot, and 12.3 °C (11.7; 12.8 °C) on cold days.

**Table 1:** Demographic and clinical characteristics of the patients

Variables	Admission after hot day (*N* = 308)	Admission after cold day (*N* = 253)	*P*
Age, years	86 (83; 89)^‡^	85 (83; 88)^‡^	0.39
Female (*N*)	57.2% (178)	58.1% (147)	0.94
Internal medicine admission	81.5% (251)	83.0% (210)	0.64
Highest socioeconomic status (*N*)	5.5% (17)	6.7% (17)	0.60
Lowest socioeconomic status (*N*)	9.7% (30)	3.6% (9)	0.004
Home care service preadmission (*N*)	36.7% (110)	28.9% (73)	0.08
Accommodation in care facility preadmission (*N*)	17.9% (55)	15.8% (40)	0.52
*Chronic conditions*			
Cerebrovascular disease (*N*)	25.6% (79)	29.2% (74)	0.34
Chronic heart failure (*N*)	44.7% (137)	48.2% (122)	0.38
Chronic kidney disease (*N*)	40.9% (126)	41.1% (104)	0.96
Dementia (*N*)	35.7% (110)	30.8% (78)	0.22
Diabetes (*N*)	28.6% (88)	32.0% (81)	0.39
Frailty (*N*)	57.5% (177)	47.8% (121)	0.02
Hypertension (*N*)	76.9% (237)	79.4% (201)	0.47
Ischemic heart disease (*N*)	37.7% (116)	44.1% (104)	0.41
Liver cirrhosis (*N*)	8.4% (26)	6.3% (16)	0.42
Malignancy (*N*)	33.4% (103)	21.3% (54)	< 0.001
*Acute conditions*			
Acute heart failure (*N*)	24.0% (74)	31.2% (79)	0.06
Acute kidney injury (*N*)	50.6% (156)	31.6% (80)	< 0.001
Hypovolemia (*N*)	52.6% (162)	21.7% (55)	< 0.001
Other causes of hyponatremia (*N*)	24.7% (76)	19.8% (50)	0.17
Fever/vomiting/diarrhea/excessive sweating (%)^‡‡^	16.6/3.9/3.2/2.3	15.0/2.4/2.8/3.2	0.55
Sepsis (*N*)	14.6% (45)	19.0% (48)	0.18
Shock (*N*)	9.4% (29)	17.8% (45)	0.004
*Medication*			
Nonsteroidal anti-inflammatory drugs (*N*)	8.1% (25)	9.5% (24)	0.81
Other drugs causing hyponatremia (*N*)	15.6% (48)	12.6% (32)	0.04
Renin-angiotensin inhibitors (*N*)	57.1% (176)	58.5% (148)	0.75
SGLT-2 inhibitors (*N*)	7.8% (24)	8.7% (22)	0.76
*Laboratory values*			
Serum sodium on admission (mmol/l)	136 (132; 139)^‡^	140 (137; 142)^‡^	< 0.001
Serum creatinine on admission (mg/dl)	1.3 (1.0; 1.8)^‡^	1.1 (0.9; 1.4)^‡^	0.04
Serum osmolality on admission (mOsm/l)	300 (291; 310)^‡^	306 (297; 315)^‡^	< 0.001
Length of stay in hospital, days	6 (4; 9)^‡^	6 (4; 8)^‡^	0.68

*SGLT‑2* sodium-glucose transporter 2

^‡^All continuous variables are expressed as medians (25th; 75th percentile)

^‡‡^Multiple entries were possible

Lowest socioeconomic status (hot vs. cold 9.7% vs. 3.6% (*N* = 30 vs. 9); *P* = 0.004), frailty (57.5% vs. 47.8% (*N* = 177 vs. 121); *P* = 0.02) and malignancy (33.4 vs. 21.3% (*N* = 103 vs. 54); *P* < 0.001) were significantly more present in patients admitted after hot days. While rates of patients admitted after hot days with “other drugs causing hyponatremia”, AKI and hypovolemia were substantially higher, lower rates were present with shock.

**Fig. 1:**
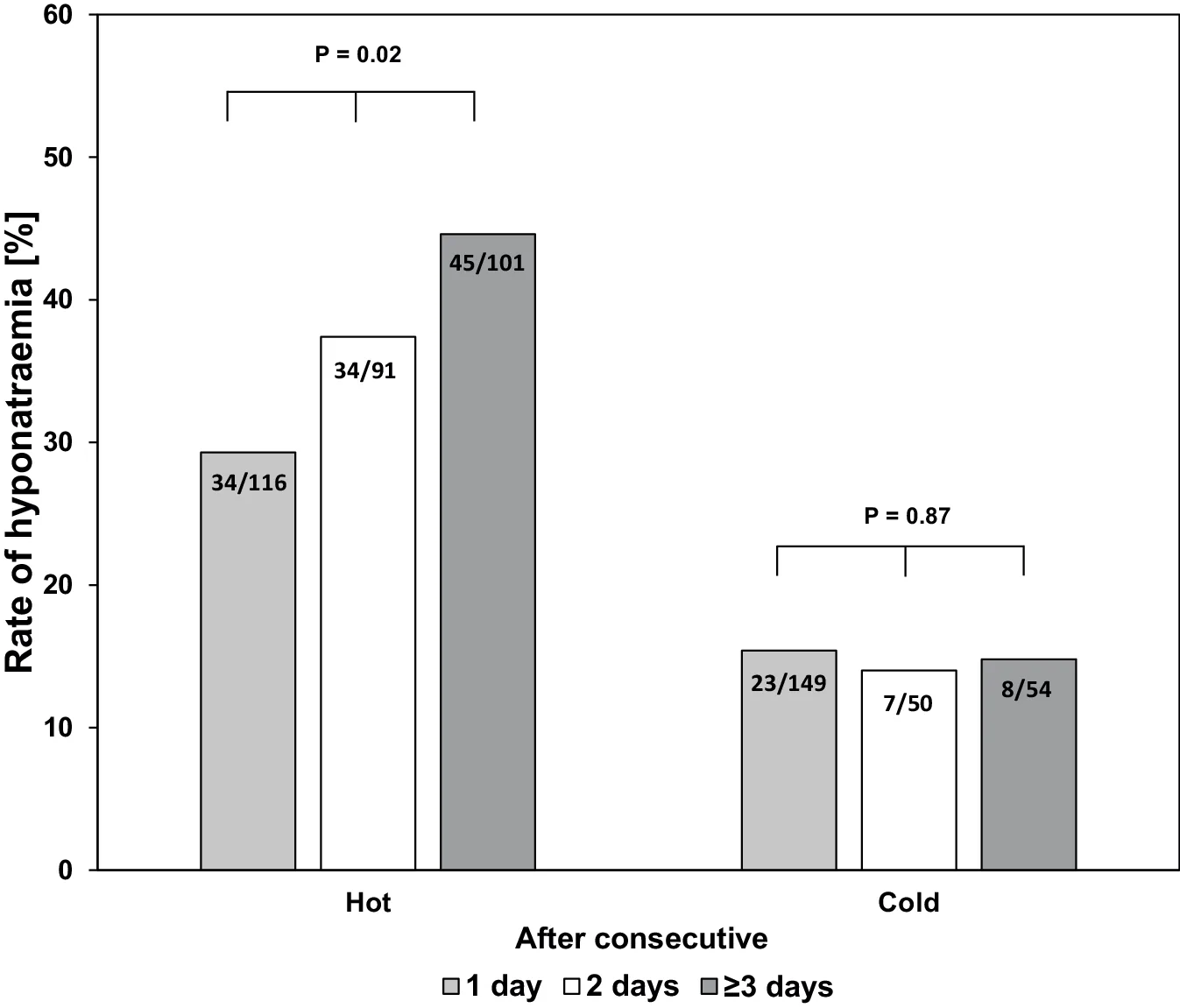
Rates of hyponatremia depending on the number of preceding consecutive hot or cold days

In the study 26.9% (*N* = 151) of the patients presented with hyponatremia. After hot days, hyponatremia was significantly more frequent compared to admissions after cold days (36.7% vs. 15.0%, *N* = 113 vs. 38; *P* < 0.001). Hyponatremia rates increased continuously and parallel to the number of consecutive hot days (Fig. 1). After exposure to ≥ 3 consecutive hot days, the hyponatremia rate exceeded that after 1 hot day by approximately 50%. In contrast, the hyponatremia rates did not differ after increasing numbers of consecutive cold days. Hyponatremia was predominately mild, acute or acute on chronic and isotonic in patients admitted after both hot and cold days (Suppl. Table 4). After hot days, there were higher frequencies of profound, moderately and severely symptomatic, and hypovolemic hyponatremia. In contrast, hyponatremia after cold days was more frequently mild, asymptomatic and associated with hypervolemia. Full, partial, marginal or no correction of hyponatremia was achieved in 65.6%, 28.7%, 4.4% and 1.3% (*n* = 99, 43, 7 and 2) of patients, respectively. The S‑Na increased from admission to day 30 by 6 mmol/l (3; 8 mmol/l). Patients admitted after hot and cold days did not differ in the degree of correction of hyponatremia (*P* = 0.35).

We identified heat as a strong risk factor for hyponatremia (Table 2). Diuretics and other causes of hyponatremia were moderately strong risk factors, frailty, hypovolemia and other drugs causing hyponatremia were weak risk factors. Accommodation in a care facility preadmission was a beneficial factor.

**Table 2:** Multivariate analysis of variables associated with hyponatremia

Variables	Adjusted odds ratios	95% Confidence limits	*P*
Male gender	1.43	0.92–2.21	0.11
Home care service preadmission	0.64	0.41–1.01	0.06
Care facility preadmission	0.24	0.13–0.44	< 0.001
Frailty	1.71	1.09–2.68	0.02
Liver cirrhosis	1.25	0.48–3.25	0.65
Diuretics	2.52	1.39–4.92	< 0.001
Other drugs causing hyponatraemia	1.82	1.12–2.98	0.02
Acute kidney injury	1.20	0.75–1.90	0.45
Other causes of hyponatraemia	2.43	1.54–4.28	0.006
Hypovolemia	1.74	1.10–2.75	0.02
Admission after hot day	4.54	2.66–7.73	< 0.001

Homer-Lemeshow test χ^2^ = 7.06, *P* = 0.50; AUROC 0.77 (0.73–0.80), *AUROC* area under the receiver operating characteristic curve (95% Confidence limits)

**Fig. 2:**
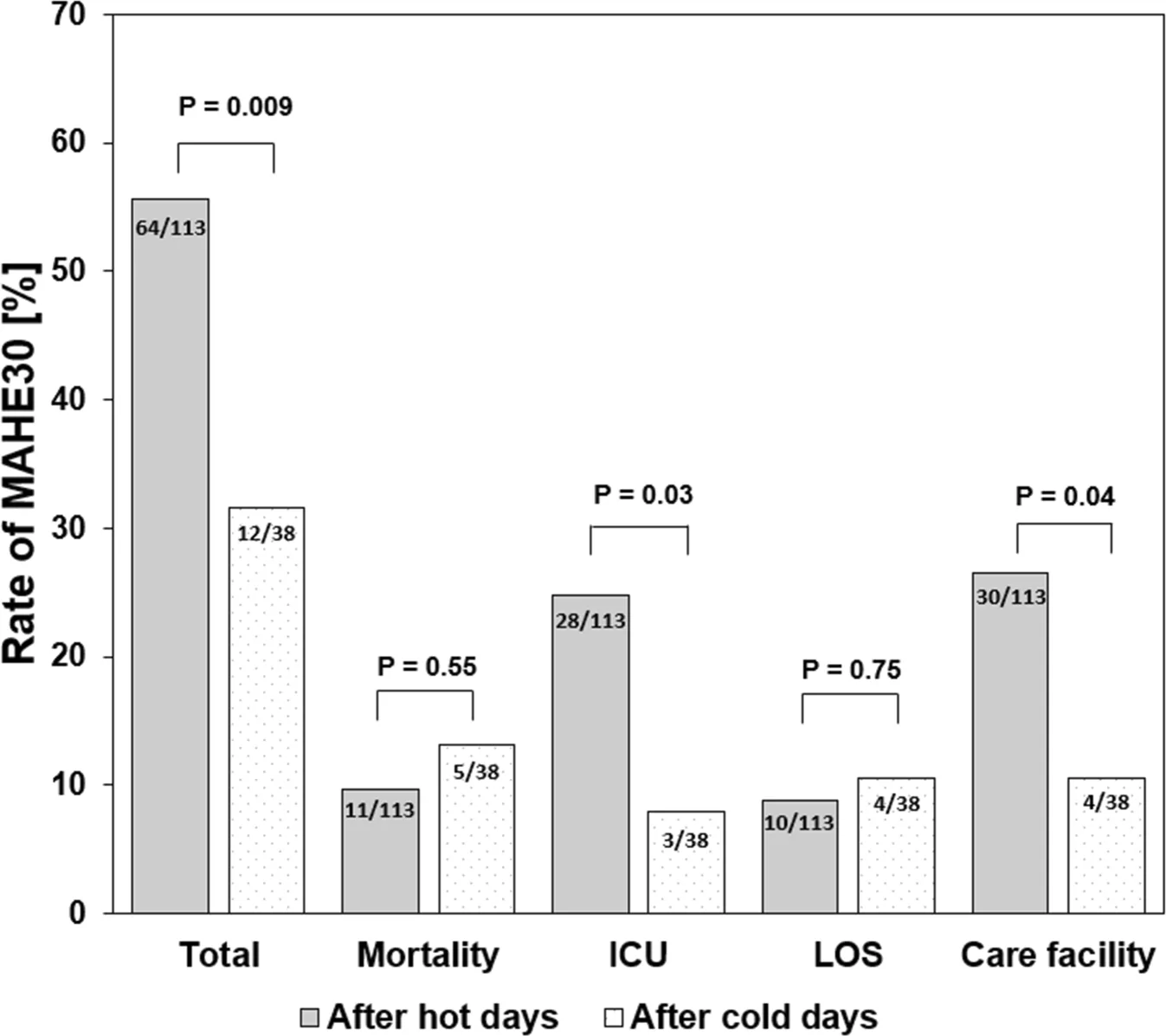
Frequencies of any major adverse hyponatremia events (MAHE30, total), and separately the components of MAHE30 mortality, admission to intensive care unit (ICU), length of stay in hospital > 90th percentile (LOS) and discharge to a care facility in patients living at home prior to admission (care facility) until day 30 after admission after hot or cold days in patients with hyponatremia

A significantly higher rate of hyponatremic patients developed MAHE30 after hot than after cold days (Fig. 2). This result was particularly determined by the higher rates of the components MAHE30^ICU^ and MAHE30^Care facility^ in patients admitted after hot days. No patient developed osmotic demyelination syndrome. Heat was identified as a strong risk factor of MAHE30 in hyponatremic patients and acute heart failure, AKI, frailty and shock as moderate risk factors (Table 3). Home care service, fully or partially corrected hyponatremia were associated with reduced likelihoods of MAHE30. Our findings in MAHE 30 were mostly confirmed by the subgroup analysis of MAHE30^ICU^ (Table 3). All regression models had high predictive power, were well fitted and left little room for variables not included to explain hyponatremia, MAHE30 or MAHE30^ICU^.

**Table 3:** Multivariate analyses of variables potentially associated with major adverse hyponatremia endpoints (MAHE30) and admission to intensive care unit until day 30 after hospital admission, both exclusively in patients with hyponatremia

	MAHE30 ^1^			Admission to intensive care unit until day 30 ^2^		
Variables	Adjusted odds ratio	95% confidence limits	*P*	Adjusted odds ratio	95% confidence limits	*P*
Low socioeconomic status	1.13	0.31–4.12	0.85	0.92	0.24–3.44	0.90
Home care service preadmission	0.30	0.14–0.65	0.003	NA	NA	NA
Dementia	1.65	0.68–3.84	0.28	0.66	0.20–2.21	0.50
Diabetes	1.41	0.58–3.45	0.45	1.85	0.66–5.16	0.24
Frailty	2.02	1.15–4.02	0.01	0.63	0.43–1.12	0.11
Ischemic heart disease	1.41	0.63–3.16	0.41	1.79	0.68–4.70	0.23
Acute heart failure	2.59	1.37–6.85	< 0.001	3.66	1.09–12.24	0.008
Acute kidney injury	1.93	1.08–5.57	0.04	1.77	1.09–3.46	0.03
Hypovolemia	1.26	0.53–3.02	0.96	NA	NA	NA
Sepsis	1.94	0.85–4.57	0.13	1.82	1.08–3.57	0.04
Shock	2.68	1.32–6.58	0.002	3.81	1.73–7.89	< 0.001
Full or partial correction of hyponatremia	0.49	0.26–0.93	0.02	NA	NA	NA
Admission after hot day	3.26	1.45–7.79	< 0.001	14.08	1.65–120.48	0.02

^1^Homer-Lemeshow test χ^2^ = 6.24, *P* = 0.62; AUROC 0.77 (0.73–0.82)

^2^Homer-Lemeshow test χ^2^ = 9.26, *P* = 0.32; AUROC 0.80 (0.75–0.86)

*NA* not applicable, *MAHE30* major adverse hyponatremia events after 30 days, *AUROC* area under the receiver operating characteristic curve

## Discussion

Our data indicate that hyponatremia occurs approximately 2.5 times more frequently in VOPs after moderate heat in Central Europe, compared to admission after cold days. This is supported by the dose-response with increasing numbers of hot days and the hyponatremia rate. We identified moderate heat as a strong risk factor of hyponatremia besides other established factors. Hyponatremic VOPs admitted after hot days carried a 75% higher burden of hyponatremia-associated morbidity and mortality compared to those admitted after cold days.

Previous investigations predominately demonstrated the effect of considerably higher ambient temperatures on hyponatremia [10, 13, 16, 17, 21]. Although there is no uniform definition of heat and the effects of absolute temperatures can differ regionally, moderate heat in northern Germany could have approximately the same effect on hyponatremia as substantially higher ambient temperatures [10, 21]. This finding is consistent with a former study [18]. Previous studies predominately used administrative data to detect hyponatremia, did not test for other explanatory variables of hyponatremia, studied patients exclusively in emergency departments, and did not correct S‑Na for glucose and other analytical interferences. While administrative data could underestimate the rates of hyponatremia, including patients admitted to other departments and correcting S-Na for analytical interferences could overestimate them [6, 17, 20]. In accordance with previous reports, our data suggest that partial or full correction of hyponatremia improves the risk of hyponatremia-associated morbidity and mortality. [2].

Our findings can have the following implications. Even when moderate heat is forecast, conditions causing hyponatremia should be corrected in VOPs and diuretics as well as other drugs causing hyponatremia paused in order to prevent hyponatremia [4, 11, 17]. Interventions should include adequate fluid intake, using electric fans or air conditioning, staying indoors and wearing cooler and sun-protective clothing also during moderate heat. General practitioners should advise frail VOP, nursing staff of home care services and care facilities, as well as family member carers about these measures and when to implement them. Response plans even for moderate heat are required, tailored in particular to VOPs [8, 14]. Although these measures seem trivial, their underuse was reported among old individuals [4, 11].

Our findings are supported by the strong statistical relationship, the temporality and dose-response of moderate heat and the development of hyponatremia, analogies to previous findings, biological plausibility, coherence and experimental evidence [10, 13, 16, 17, 21]. Hyponatremia itself is of little relevance as an outcome per se [1, 9, 20, 22]. One strength is our finding that MAHE30, a bundle of clinically relevant adverse effects of hyponatremia, can be a meaningful outcome, in hyponatremic VOPs exposed to heat; however, MAHE30 is newly constructed and requires vigorous validation. Thirdly, we optimized internal validity and substantially reduced potential confounding and bias by restricting our study to patients of interest, exact definitions, comprehensive search, inclusion and identical measurements of variables and outcomes, and correction for differences by multivariate regression. Fourthly, sodium measurement by direct potentiometry and its adjustment for glucose, permitted correct determination of hyponatremia, similar to only one study [16, 20]. Further strengths are the 30-day follow-up of a medium-sized patient cohort with a typical cross-section of VOPs [3, 13]. Major limitations of the study are the single-center and observational design examining the effect at the extremes of the temperature range in especially vulnerable individuals. Additionally, we studied only one large city in northern Germany, which is considered one of the greenest ones in Germany. It is further substantially limiting that no data were available on individual exposure to hot and cold temperature, access to air conditioning, fluid intake and time spent outdoors. Our findings that patients admitted after hot days more frequently came from the lowest socioeconomic background and suffered from frailty and malignancies may indicate their limited access to support and resources which permit adequate adaptation to heat. Additionally, frailty and malignancies are both associated with reduced physiological reserves and increased vulnerability to heat. Our results remain hypothesis generating and require validation in larger, multicenter studies including different urban and rural areas, all ages and temperatures to permit generalizability. Heat-associated hyponatremia will more likely occur in the future in VOPs and require considerable medical and economic resources [1, 5, 7, 22]. Hence, it seemed reasonable to study particularly these patients.

## Conclusion and practical recommendations

Moderate heat is highly associated with hyponatremia in VOPs and is its strong risk factor. After hot days, hyponatremic VOPs are at a substantially increased risk for severe morbidity and mortality of hyponatremia. Besides the

- development of tailored response plans for moderate heat

we recommend

- to correct all modifiable conditions causing hyponatremia
- to pause diuretics and other drugs causing hyponatremia
- to consequently implement all individual preventative measures

in VOP when moderate heat is forecast.

## Supplementary Information

ESM1: Supplementary material 1

## Data Availability

The dataset of this study will be shared on request to the corresponding author.

## Declarations

All procedures in this study are in accordance with the 1964 Helsinki declaration. The study protocol was approved by the local ethics committee (Medical Council Bremen, HR/RE-877).
