# Supplementary material for: Heat-associated hyponatremia and its outcomes in very old inpatients in northern Germany
Source: Z Gerontol Geriatr. 2026 Sep 2;59(6):508–14. doi: 10.1007/s00391-026-02635-5 (PMC13597654; doi:10.1007/s00391-026-02635-5)
Supplement: Supplementary file 1 [file 391_2026_2635_MOESM1_ESM.docx]

**SUPPLEMENTARY MATERIAL**

**Supplementary table 1**. Definitions of variables

| **Variables** | **Definitions** |
| --- | --- |
| Accommodation in care facility | Patient living in a retirement home as documented in the electronic hospital records |
| Acute heart failure | Established chronic heart failure or de-novo acute heart failure with new or worsening signs and symptoms such as rapid weight gain, exertional dyspnoea, orthopnoea, dependent and pulmonary oedema, elevated jugular venous pressure, and peripheral hypoperfusion, and characteristic findings in echocardiography, chest X-ray, lung ultrasound, plasma NT-proBNP and others [1] |
| Acute kidney injury | Increase or decrease in serum creatinine (SCr), either by 0.3 mg/dl in 48 hours or 1.5 – 1.9 times from baseline SCr within ≤ 7 days, and/or decline of urine output (UO) < 0.5 ml/kg/h or UO increase ≥ 0.5 ml/kg/h, both for at least 6 hours [2-4] |
| Chronic kidney disease | Estimated glomerular filtration rate in the range of 15 to 44 ml/min/1.73 m^2^ determined by at least 2 measurements in at least 3 months, known as stages G3b and G4 [5] |
| Correction of hyponatraemia   - complete - partial - marginal - none | Serum sodium (S-Na) last measured within 30 d after admission  ≥ 135 mmol/l  130 – 134 mmol/l  129 – 120 mmol/l  ≤ 119 mmol/l |
| Definitions of other chronic conditions | Cerebrovascular disease, chronic heart failure, dementia, ischaemic heart disesase and liver cirrhosis were defined by diagnoses recorded in the electronic hospital records |
| Diabetes | (1) Diabetes documented prior to ICU admission, (2) fasting venous glucose ≥ 126 or capillary glucose ≥ 110, and/or random venous glucose ≥ 200 mg/dl, each on two separate tests, (3) HbA1c value ≥ 6,5%, and/or (4) use of antidiabetic medication prior to this study [6] |
| Diuretics | Administration of thiazide and/or loop diuretics prior to hospital admission |
| Estimated glomerular filtration rate (eGFR) | Estimation by the European Kidney Function Consortium equation [7] |
| Frailty | Frailty was defined by clinical frailty scores ≥ 5 [8] |
| Home care service | Existence of outpatient nursing service as documented in the electronic hospital records |
| Hypertension | Arterial blood pressure ≥ 140/90 mm Hg requiring antihypertensive medication |
| Hyponatraemia – measurement | S-Na was measured by direct potentiometry using blood gas analyzers in undiluted, whole blood to avoid pseudohyponatraemia caused by monoclonal gammopathy and elevated serum lipids and protein [9] |
| Hyponatraemia – equation to adjusted for blood glucose | S-Na was adjusted for blood glucose > 100 mg/dl by the equation: Adjusted S-Na [mmol/l] = measured S-Na [mmol/l] + (2.4 x blood glucose [mg/dl] – 100)/100 [10] |
| Hyponatraemia – severity and subtypes | The following classifications of hyponatraemia are based upon serum sodium concentration, symptoms, duration of develop-ment, serum osmolality and volume status, according to [10] |
| Hyponatraemia   - mild - moderate - profound | Serum sodium (S-Na) on admission  130 – 134 mmol/l  125 – 129 mmol/l  ≤ 124 mmol/l |
| Hyponatraemia   - subsymptomatic - moderately symptomatic - severely symptomatic | S-Na < 135 mmol/l on admission and  no new symptoms or findings as described below  forgetfulness, gait disturbance, headache, lethargy, nausea, vomiting, weakness ^1^  cardiorespiratory arrest, cognitive impairment, coma, confusion, Glasgow coma scale < 12, seizure ^1^  ^1^ all symptoms and signs without obvious other causes than hyponatraemia |
| Hyponatraemia   - acute - acute-on-chronic - chronic | S-Na < 135 mmol/l on admission for  ≤ 48 h pre- or after admission with preceding normal S-Na  > 48 h with further decrease of S-Na in ≤ 48 h pre- or after admission  > 48 h or when no preadmission S-Na was documented |
| Hyponatraemia   - hypotonic - isotonic - hypertonic | S-Na < 135 mmol/l on admission and  calculated serum osmolality < 275 mOsm/l  calculated serum osmolality 275 – 295 mOsm/l  calculated serum osmolality > 295 mOsm/l |
| Hyponatraemia   - hypovolaemic - euvolaemic - hypervolaemic | S-Na < 135 mmol/l on admission and inspiratory collapsibility of the inferior vena cava compared to expiratory diameter  ≥ 50 %  49 – 15 %  < 15 % |
| Hypovolaemia | Hypovolaemia was defined as manually assessed axillary dryness, reduced skin turgor, systolic blood pressure < 100 mm Hg and/or inspiratory collapsibility of the inferior vena cava ≥ 50 % compared to expiratory diameter [11]. |
| Malignancy | Any active malignancy documented in the electronic hospital records |
| Non-steroidal anti-inflammatory drug | Administration of any non-steroidal anti-inflammatory drug or cyclooxygenase-2 inhibitor |
| Sepsis | Definition according to [12] |
| Serum osmolality | Calculation by the following equation [13]:  S-Osm [mOsm/l] = 1.86 x (S-Na [mmol/l] + serum potassium [mmol/l]) + 1.15 x (0.0555 x blood glucose [mg/dl]) + (0.167 x serum urea [mg/dl]) + 14 |
| Shock | Systolic blood pressure < 90 mm Hg for > 30 minutes requiring vasopressors |
| Socioeconomic status | Determination in accordance with the Bremen social deprivation-index from 2009 which is based on census and public data on education, income, unemployment, social benefits, poverty rate, crime statistics and immigration flow, utilizing postal codes of home addresses, modified by data from 2021. It was stratified in quartiles according to the following postal codes (high: 28209, 28211, 28213, 28355, 28359, 28759; intermediate high: 28205, 28215, 28217, 28219, 28279, 28309, 28357, 28717, 28777; intermediate low: 28197, 28199, 28201, 28207, 28237, 28259, 28277, 28307, 28327, 28755, 28757, 28779; low: 28195, 28239, 28325) [14, 15] |
| Strength of association | Strength of association was classified as strong (adjusted odds ratio (aOR) ≥ 3.0), moderate (aOR 1.86 – 2.99) or weak (aOR ≤ 1.85) [16]. |

Diuretics, non-steroidal anti-inflammatory drugs, renin-angiotensin-inhibitors, sodium-glucose co-transporter-2-inhibitors, ‘other drugs causing hyponatraemia’, and ‘other conditions associated with hyponatraemia’ were considered as risk factors of hyponatraemia when they were administered or occurred at least 3 days preceding admission to hospital.

**REFERENCES**

1. McDonagh TA, Metra M, Adamo M, et al. (2021) ESC Guidelines for the diagnosis and treatment of acute and chronic heart failure: Eur Heart J 42:3599-3726
2. Kellum JA, Lameire N, Aspelin P et al. (2012) KDIGO clinical practice guideline for acute kidney injury. Kidney Int Suppl 2:S1–S138
3. Macedo E, Hemmila U, Sharma SK et al (2021) Recognition and management of community-acquired acute kidney injury in low-resource settings in the ISN 0by25 trial: A multi-country feasibility study. PLoS Med 18:e1003408
4. Wainstein M, MacDonald S, Fryer D et al (2022) Use of an extended KDIGO definition to diagnose acute kidney injury in patients with COVID-19: A multinational study using the ISARIC-WHO clinical characterisation protocol. PLoS Med 19:e1003969
5. Levey AS, de Jong PE, Coresh J et al (2011) The definition, classification, and prognosis of chronic kidney disease: a KDIGO Controversies Conference report. Kidney Int 80:17–28
6. American Diabetes Association <https://www.diabetes.org/diabetes/a1c/diagnosis> (last accessed 24.10.25)
7. Pottel H, Björk J, Courbebaisse M et al (2021) Development and validation of a modified full age spectrum creatinine-based equation to estimate glomerular filtration rate: a cross-sectional analysis of pooled data. Ann Intern Med 174:183-191
8. Stille K, Temmel N, Hepp J, Herget-Rosenthal S (2020) Validation of the clinical frailty scale for retrospective use in acute care. Eur Geriatr Med 11:1009-1015
9. Katrangi W, Baumann NA, Nett RC, Karon BS, Block DR (2019) Prevalence of clinically significant differences in sodium measurements due to abnormal protein concentrations using an indirect Ion-selective electrode method. J Appl Lab Med 4:427–432
10. Spasovski G, Vanholder R, Allolio B et al (2014) Clinical practice guideline on diagnosis and treatment of hyponatraemia. Nephrol Dial Transplant Suppl 2:i1-i39
11. Deißler L, Wirth R, Frilling B, Janneck M, Rösler A (2023) Hydration status assessment in older patients. Dtsch Arztebl Int 120:663-669
12. Singer M, Deutschman CS, Seymour CW, et al (2016) The third international consensus definitions for sepsis and septic shock. JAMA 315:801-810
13. Hooper L, Abdelhamid A, Ali A et al (2015) Diagnostic accuracy of calculated serum osmolarity to predict dehydration in older people: adding value to pathology laboratory reports. BMJ Open 5:e008846
14. Schmucker J, Seide S, Wienbergen H et al (2017) Socially disadvantaged city districts show a higher incidence of acute ST-elevation myocardial infarctions with elevated cardiovascular risk factors and worse prognosis. BMC Cardiovasc Disord 17:254
15. Senatorin für Soziales, Jugend, Integration und Sport (Ed.) (2021) Lebenslagen im Land Bremen. 3. Bericht des Senats der Freien Hansestadt Bremen 2021. <https://www.soziales.bremen.de/sixcms/media.php/13/Dritter%20Lebenslagenbericht%20Land%20Bremen.pdf> (last accessed 14 March 2026)
16. Olivier J, May WL, Bell ML (2017) Relative effect sizes for measures of risk. Commun Stat Theor Methods 46:6774–6781

**Supplementary table 2.** Bivariate analyses of variables potentially associated with hyponatraemia

| **Variables** | **Risk ratio** | **95 % Confidence limits** | **P** |
| --- | --- | --- | --- |
| Male gender | 1.37 | 1.02 – 1.83 | 0.04 |
| High socioeconomic status | 0.89 | 0.48 – 1.65 | 0.70 |
| Low socioeconomic status | 1.42 | 0.73 – 2.77 | 0.30 |
| Home care service preadmission | 0.76 | 0.56 – 0.98 | 0.03 |
| Care facility preadmission | 0.63 | 0.46 – 0.74 | 0.003 |
| **Chronic conditions** |  |  |  |
| Cerebrovascular disease | 1.23 | 0.92 – 1.65 | 0.17 |
| Chronic heart failure | 1.14 | 0.87 – 1.49 | 0.37 |
| Chronic kidney disease | 1.13 | 0.82 – 1.29 | 0.48 |
| Dementia | 1.07 | 0.81 – 1.43 | 0.62 |
| Diabetes | 1.19 | 0.84 – 1.38 | 0.54 |
| Frailty | 1.47 | 1.12 – 1.99 | 0.007 |
| Hypertension | 0.94 | 0.84 – 1.04 | 0.22 |
| Ischaemic heart disease | 1.13 | 0.85 – 1.51 | 0.40 |
| Liver cirrhosis | 1.34 | 1.02 – 1.93 | 0.04 |
| Malignancy | 1.04 | 0.76 – 1.45 | 0.80 |
| **Acute conditions** |  |  |  |
| Acute heart failure | 1.13 | 0.84 – 1.51 | 0.43 |
| Acute kidney injury | 1.25 | 1.02 – 1.53 | 0.03 |
| Hypovolaemia | 2.01 | 1.52 – 2.65 | < 0.001 |
| Other causes of hyponatraemia | 1.59 | 1.06 – 2.39 | 0.03 |
| Sepsis | 1.27 | 0.84 – 1.93 | 0.26 |
| Shock | 1.25 | 0.79 – 1.98 | 0.33 |
| **Medication** |  |  |  |
| Diuretics | 2.70 | 1.79 – 5.43 | < 0.001 |
| Non-steroidal anti-inflammatory drugs | 1.18 | 0.79 – 1.84 | 0.47 |
| Other drugs causing hyponatraemia | 1.21 | 0.98 – 1.50 | 0.08 |
| Renin-angiotensin-inhibitors | 1.10 | 0.83 – 1.46 | 0.50 |
| SGLT-2 inhibitors | 0.32 | 0.05 – 2.28 | 0.28 |
| Admission after hot day | 2.65 | 1.89 – 3.73 | < 0.001 |

SGLT-2 - Sodium-glucose co-transporter-2.

**Supplementary table 3.** Bivariate analyses of variables potentially associated with major adverse hyponatraemia endpoints (MAHE30) and admission to intensive care unit both until day 30 after hospital admission exclusively in patients with hyponatraemia

|  | **MAHE30** | | | **Admission to intensive care unit until day 30** | | |
| --- | --- | --- | --- | --- | --- | --- |
| **Variables** | **Risk ratio** | **95 % Confidence limits** | **P** | **Risk ratio** | **95 % Confidence limits** | **P** |
| Male gender | 1.10 | 0.78 – 1.56 | 0.57 | 1.52 | 0.30 – 2.84 | 0.19 |
| High socioeconomic status | 0.99 | 0.49 – 2.02 | 0.98 | 0.63 | 0.39 – 1.36 | 0.41 |
| Low socioeconomic status | 3.62 | 1.05 – 12.46 | 0.04 | 1.88 | 0.96 – 3.92 | 0.07 |
| Home care service preadmission | 0.58 | 0.42 – 0.79 | < 0.001 | 0.77 | 0.41 – 1.45 | 0.43 |
| Cerebrovascular disease | 1.22 | 0.89 – 1.68 | 0.22 | 1.20 | 0.62 – 2.31 | 0.55 |
| Chronic heart failure | 1.16 | 0.84 – 1.59 | 0.37 | 1.11 | 0.59 – 2.08 | 0.74 |
| Chronic kidney disease | 1.26 | 0.92 – 1.72 | 0.16 | 1.09 | 0.58 – 2.05 | 0.79 |
| Dementia | 1.48 | 0.95 – 2.32 | 0.08 | 0.62 | 0.30 – 1.30 | 0.08 |
| Diabetes | 1.37 | 1.002 – 1.88 | 0.048 | 1.69 | 0.98 – 3.19 | 0.06 |
| Frailty | 1.32 | 1.03 – 1.68 | 0.02 | 0.45 | 0.24 – 0.83 | 0.01 |
| Hypertension | 1.16 | 0.79 – 1.71 | 0.45 | 1.03 | 0.51 – 2.13 | 0.92 |
| Ischaemic heart disease | 1.34 | 0.98 – 1.83 | 0.06 | 1.44 | 1.05 – 2.27 | 0.01 |
| Liver cirrhosis | 1.35 | 0.83 – 2.21 | 0.22 | 2.21 | 0.49 – 6.68 | 0.39 |
| Malignancy | 1.16 | 0.79 – 1.69 | 0.45 | 0.55 | 0.24 – 1.24 | 0.15 |
| Acute heart failure | 1.47 | 1.08 – 1.99 | 0.01 | 2.42 | 1.29 – 4.55 | 0.006 |
| Acute kidney injury | 1.60 | 1.15 – 2.22 | 0.006 | 2.15 | 1.17 – 3.96 | 0.01 |
| Hypovolaemia | 1.51 | 0.98 – 2.63 | 0.06 | 1.26 | 0.10 – 16.13 | 0.86 |
| Other causes of hyponatraemia | 1.33 | 0.73 – 2.44 | 0.35 | 1.20 | 0.49 – 3.06 | 0.67 |
| Sepsis | 2.25 | 1.67 – 3.25 | < 0.001 | 2.68 | 1.43 – 4.92 | < 0.001 |
| Shock | 2.72 | 1.88 – 4.14 | < 0.001 | 3.31 | 1.28 – 7.43 | < 0.001 |
| Full or partial correction of hyponatraemia | 0.67 | 0.53 – 0.94 | 0.03 | 0.88 | 0.46 – 1.67 | 0.70 |
| Admission after hot day | 1.79 | 1.09 – 2.94 | 0.02 | 10.09 | 1.42 – 71.49 | 0.009 |

**Supplementary table 4.** Severity and subtypes of hyponatraemia on admission after hot and cold days

|  | **Hot days** | **Cold days** | **P** |
| --- | --- | --- | --- |
|  | N = 308 | N =253 |  |
| Mild hyponatraemia | 61 (54.0 %) | 28 (73.7 %) | 0.01 |
| Moderate hyponatraemia | 29 (25.7 %) | 8 (21.0 %) |  |
| Profound hyponatraemia | 23 (20.3 %) | 2 (5.3 %) |  |
| Asymptomatic hyponatraemia | 29 (25.7 %) | 17 (44.7 %) | 0.03 |
| Moderately symptomatic hyponatraemia | 54 (47.8 %) | 15 (39.5 %) |  |
| Severely symptomatic hyponatraemia | 30 (26.5 %) | 6 (15.8 %) |  |
| Acute hyponatraemia | 45 (39.8 %) | 14 (36.8 %) | 0.55 |
| Acute-on-chronic hyponatraemia | 50 (44.3 %) | 16 (42.1 %) |  |
| Chronic hyponatraemia | 18 (15.9 %) | 8 (21.1 %) |  |
| Hypotonic hyponatraemia | 24 (21.2 %) | 9 (23.7 %) | 0.45 |
| Isotonic hyponatraemia | 57 (50.5 %) | 21 (55.3 %) |  |
| Hypertonic hyponatraemia | 32 (28.3 %) | 8 (21.0 %) |  |
| Hypovolaemic hyponatraemia | 68 (60.2 %) | 16 (42.1 %) | 0.02 |
| Euvolaemic hyponatraemia | 32 (28.3 %) | 12 (31.6 %) |  |
| Hypervolaemic hyponatraemia | 13 (11.5 %) | 10 (26.3 %) |  |

**Supplementary figure.** Flow diagram of the study participants.

**Identification**

Patients ≥ 80 years admitted to hospital 2011 – 2023 (N = 33695)

**Exclusion**

Analysed (N = 561)

**Analysis**

**Inclusion**

Included in the study (N = 561)

- Chronic kidney disease stage G5 (N = 19)
- Missing data (N = 18)
- Decision on admission to withhold life-sustaining treatment (N = 23)

Patients ≥ 80 years admitted unplanned to the Dept. of Medicine and Trauma surgery 01.06.-31.08., 2011-2023 on hot or cold days (N = 621)

Admitted unplanned to the Dept. of Medicine and Trauma surgery 01.06.-31.08., 2011-2023 neither on hot nor cold days (N = 4658)

Patients ≥ 80 years admitted unplanned to the Dept. of Medicine and Trauma surgery 01.06.-31.08., 2011-2023 (N = 5279)

Admitted unplanned to the Dept. of Medicine and Trauma surgery 01.01.-31.05. and 01.09.-31.12., 2011-2023 (N = 16806)

Patients ≥ 80 years admitted unplanned to the Dept. of Medicine and Trauma surgery (N = 22085)

- Admitted to other departments (N = 10107)
- Planed admission to the Departments of Medicine and Trauma surgery (N = 1503)
